# Supplementary material for: Only Small Effects of Mindfulness-Based Interventions on Biomarker Levels of Inflammation and Stress: A Preregistered Systematic Review and Two Three-Level Meta-Analyses
Source: Int J Mol Sci. 2023 Feb 23;24(5):4445. doi: 10.3390/ijms24054445 (PMC10003032; doi:10.3390/ijms24054445)
Supplement: Supplementary file 1 [file ijms-24-04445-s001.zip › ijms-2229960-supplementary.pdf]

Supplementary Material

Table S1

Mindfulness-Based Interventions Used in Primary Studies

**MBSR – Mindfulness-based stress reduction**<br>MBSR is an eight-week course program with weekly group meetings of 2.5 hours, where different types of mindful meditation forms are taught and practiced. Those practices include sitting meditation with a focus on breathing, body scan, mindful yoga exercise and walking meditation. The goal of this course is to integrate mindfulness in the participants daily life. During the duration of the course, participants are obligated to practice different types of mindful meditation at home (60min daily). Between week six and seven, an intensive mindfulness day is held, where participants practice about 7 hours of different meditation practices (Kabat-Zinn, 1990).
**MBCT – Mindfulness-based cognitive therapy**<br>MBCT is an eight-week course program with weekly group meetings, which includes different elements of mindfulness meditation practices (comparable to the MBSR program) as well as cognitive therapy elements. On the one hand, the goal of MBCT is to apply mindfulness in the participants daily life and the other hand, it is about gaining a better understanding of the working of the mind, coping with stress and negative emotions/thoughts. During the duration of the course, participants are obligated to practice different types of mindful meditation at home (60min daily). Towards the end of the course an intensive practice day is held, where participants practice several hours of different mindful meditation practices (Segal et al., 2002).
**MBRP – Mindfulness-based relapse prevention**<br>MBRP is an eight-week program with weekly group sessions of 2 hours, during which participants with a history of substance use disorders learn formal mindfulness practices as well as how to integrate them into their daily life. In MBRP the focus lies especially on the awareness for situations that create a high risk for potential relapse and on methods that help coping with the craving for a certain substance. During the course participants are obligated to practice different types of mindful meditation at home (Witkiewitz et al., 2005).
**HEM – Health enhancement through mindfulness**<br>HEM is a residential intensive three-day course based on activities of the MBSR program, like sitting and walking meditation, body scan, mindful eating, mindful stretching and discussions (Creswell et al., 2016).
**MAP – Mindful awareness practice for daily living**<br>MAP is a six-week program with weekly group sessions of 2 hours developed by the the Mindful Awareness Research Center (MARC). Classes include different types of mindful meditation like sitting meditation, walking or standing meditation, eating meditation as well as methods that help deal with emotions and pain. During the duration of the course participants are asked to do daily meditation homework, staring with 5min per day up to 20min per day (Black et al., 2015).
**bMM – Brief mindfulness meditation**<br>bMM is a three-day mindfulness course, during which participants practice 30min of sitting meditation with an audio guide which is based on the standard sitting meditation of MBSR (Sousa et al. 2021).

**Mindful Parenting**<br>Mindful Parenting is a six-week course with weekly group sessions of 1.5 hours that included typical mindfulness practices of the MBSR program. Further, the program focuses on different elements and difficulties of parenting (Ho et al., 2020).
**Mindfulness Matters**<br>Mindfulness Matters is an eight-week course with weekly sessions of 1 hour, especially designed for children between five and eight years old. The course includes short mindful meditation practices as well as methods like storytelling. The parents and kids get weekly audio files to practice at home (Ho et al., 2020).
**ACT – Acceptance and Commitment Therapy**<br>Here ACT is practiced as an eight-week program with a total of six group sessions of 1.5 hours. The sessions include mindfulness-based elements and focus on one´s values, committing to those values and general acceptance and flexibility. Besides the group sessions, participants are asked to practice at home using a personal workbook (Järvelä-Reijonen et al, 2020).
**MMFT – Mindfulness-based mind fitness training**<br>MMFT is an eight-week course with weekly group sessions of 2 hours and an additional intensive practice workshop of 4 hours. Besides the class sessions participants are obligated to practice a minimum of 30min of mindfulness exercises and self-regulation practices. It focuses on the development of control and tolerance of challenging experiences and stress resilience (Johnson et al., 2014).
**PCbMP – Primary care brief mindfulness practice**<br>PCbMP is a four-week program with weekly group sessions of 1.5hours. The program includes typical mindful meditation elements of MBSR and has a special focus on the enhancement of emotional regulation and cognitive reappraisal of stressors. Besides the group sessions, participants are asked to practice with the audio files of MBSR (Bergen-Cico et al., 2014).
**MBX – Mindfulness-based stretching and deep breathing exercise**<br>MBX consists of 16 semiweekly group trainings of 1 hour. The intervention has its focus on balancing and stretching movements combined with deep breathing and mindfulness (Kim et al., 2013).
**MBI – Monitoring and accepting**<br>This MBI is an eight-week long course with weekly group sessions of 2 hours and follows the standard MBSR protocol with a specific focus on monitoring and acceptance. Besides the group sessions, participants are asked for daily 45min home practices (Villaba et al., 2019).
**MBI – MA App**<br>This smartphone-based MBI is a two-week long program with daily 20min audio-guided practices and additional 3-10min of none-guided practice. It focuses on the monitoring and accepting the body and emotions and is based on elements of MBSR (Villaba et al., 2019).
**MMP Heart and Mind – Mindful meditation program: healing the heart and mind**<br>This program is an eight-week course with weekly group sessions of 2 hours and combines main elements of MBSR as well as elements of cognitive therapy. The content of the the classes was especially adapted fit the needs of health-care workers. During the duration of

the course participants are obligated to practice 30min a day with audio recordings (Galantino et al., 2005).
**MBSR-T – Mindfulness-based stress reduction for teenagers**<br>MBSR-T is a four-week program with 8 semiweekly group sessions, especially adapted for adolescents (13-18 years). Compared to the MBSR program the formal mindful meditation practices are shortened (10-20min instead of 45min) and slightly different vocabulary is used ("on-your-own-practice" instead of homework) (Biegel et al., 2009).
**MBI – Workplace**<br>This program is an eight-week course with 8 weekly group sessions of 1 hour, especially designed to be used in workplace settings. It combines elements of mindfulness meditation, yoga and relaxation through music. If possible, participants should practice daily for 20min. (Duchemin et al.,2015).
**MBI – Physicians**<br>This protocol is an eight-week program with 8 weekly group sessions, very similar to the MBSR course, but tailored to physician's workplace needs (Fendel et al., 2020).
**MBI – Smokers**<br>MTS is mindfulness training for smokers, basically following the MBSR program with a special focus on difficulties that people that want to quit smoking face (Davis et al., 2007).
**MBI – Training**<br>This MBI is an eight-week course with weekly group workshop sessions of 1 hour and contains elements of MBSR and MBCT. Besides the weekly group sessions, it further included daily 30min of home practice and mindful Wushu training sessions. The aim is to allow the adaption of mindfulness into the participants competitive environment and sport practices (Mehrsafar et al., 2019).
**MBI – Dementia caregivers**<br>This MBI is a six-week program with weekly group sessions of 1.5 hours and combines elements of MBSR and MBCT. The program is adapted to fit the specific needs and difficulties of dementia caregivers and focuses on different aspects of stress and to promote self-care as well as feelings of competence and mastery. Further participants are asked to practice at home daily (Oken et al., 2010).
**MBI – Audio**<br>This MBI uses a 20min audio guide version of the MBSR body scan. Participants are asked to practice daily for the duration of 8 weeks (Schultchen et al., 2019).
**MBI – University**<br>This MBI lasts for three-month and is embedded in a standard curriculum course for medical students and includes a one-day introduction, four 3-hour group sessions and a final 2 hour closing session. Students learn and practice about typical elements of the MBSR program and are asked to practice daily (Stoffel et al., 2019).
**MBI – Students**<br>This MBI is a seven-week program with weekly group sessions of 1.5 hours and is based on the mindfulness course book by Williams & Pennman (2011). It is adapted for students and focuses on flexibility, self-compassion and empowerment to optimize wellbeing and resilience (Williams & Pennmann, 2011).
**MBI – Teachers**

This MBI is a 16-week program with one monthly 1.5 hours group meeting, especially designed to reduce stress and burnout of teachers. Besides the group sessions, participants become workbooks for their home practice (Taylor et al., 2021).

Note. Full references are provided in the reference list in the main text.

Table S2

Inclusion and Exclusion Criteria

Variables	Inclusion Criteria	Exclusion Criteria
Participants	With psychiatric diagnosis or at-risk, stressed, or healthy subjects	Physical illness
Interventions	MBIs	Other interventions
Outcome	Inflammatory/stress biomarkers	No inflammatory/stress biomarker
Study Design	RCTs, non-RCTs, and single-arm or multiple-arm trials with pre/posttest scores	Qualitative studies
Publication	Full-text articles and grey literature	Reviews, case reports, letters, and conference abstracts

Note. MBIs = mindfulness-based intervention; RCTs = randomized controlled trials; non-RCTs = non-randomized controlled trials.

Table S3

List of Search Terms That Were Used for the Systematic Literature Search

Mindfulness	MBIs	Sample	Outcome
Mindful*	MBSR	Psychiatric disorder*	Biomarker*
Meditation*	Mindfulness Based Stress Reduction	Psychiatric*	Low-grade inflammation
	MBCT	Psychiatric diagnose*	Neuroinflammation
	Mindfulness-Based Cognitive Therapy	At-risk	Biological markers
	MBRP	Stress*	Inflammation*
	Mindfulness-Based Relapse Prevention	Healthy	Neuropeptide*
	MBI*	Physical illness	Cytokine*
	Mindfulness-Based Intervention*	Depression*	C-reactive protein*
	ACT		CRP
		Sleep disorder*	IL-6
		Insomnia	IL-8
		Substance use disorder*	IL-4
		Addiction*	IL-10
		PTSD	TNF-α
		ADHD	IFN-γ
		Anxiety	Cortisol
			Chemokine*
			ACTH
			BDNF

Note. All search terms of the four categories were combined Boolean operators. The asterisk (*) represents variable endings of a root word.

Table S4

Calculation of Effect Sizes for Studies That did not Report Sufficient Data

Calculation	Study
Means and standard deviations based on medians and ranges	Gex-Fabry et al. (2012)<br>Järvelä-Reijonen et al. (2020)<br>Meyer et al. (2019)
Hedges *g* from *t* values	Duchemin et al. (2015)<br>Galantino et al. (2005)
Hedges *g* from regression analysis	Creswell et al. (2014)<br>Stoffel et al. (2019)
Hedges *g* from ANOVA table	Ramler et al. (2015)<br>Nyklicek et al. (2013)
Hedges *g* from Cohen *d*	Bellosta-Batalla et al. (2020)<br>Brand et al. (2012)<br>Eisendrath et al. (2016)<br>Johnson et al. (2014)<br>Mehrsafar et al. (2019)

Note. Full references are provided in the reference list in the main text.

Table S5

Studies Reporting Follow-Up Data

Studies	Length of Follow-Up	Included in
Järvelä-Reijonen et al. (2020)	36 weeks	TLMA 1 + 2
Mehrsafar et al. (2020)	8 weeks	TLMA 2
Meyer et al. (2019)	17 weeks	TLMA 1 + 2
Ng et al. (2020)	16 weeks	TLMA 1 + 2
Roberts et al. (2020)	24 weeks	TLMA 1
Walsh et al. (2016)	12 weeks	TLMA 1

Note. Full references are provided in the reference list in the main text.
